# A Day in the Life of the Exon Junction Complex

**DOI:** 10.3390/biom10060866

**Published:** 2020-06-05

**Authors:** Lena P. Schlautmann, Niels H. Gehring

**Affiliations:** Institute for Genetics, University of Cologne, Zuelpicher Str. 47a, 50674 Cologne, Germany; lschlau2@uni-koeln.de

**Keywords:** mRNA, gene regulation, RNA processing, RNA-binding protein

## Abstract

The exon junction complex (EJC) is an abundant messenger ribonucleoprotein (mRNP) component that is assembled during splicing and binds to mRNAs upstream of exon-exon junctions. EJCs accompany the mRNA during its entire life in the nucleus and the cytoplasm and communicate the information about the splicing process and the position of introns. Specifically, the EJC’s core components and its associated proteins regulate different steps of gene expression, including pre-mRNA splicing, mRNA export, translation, and nonsense-mediated mRNA decay (NMD). This review summarizes the most important functions and main protagonists in the life of the EJC. It also provides an overview of the latest findings on the assembly, composition and molecular activities of the EJC and presents them in the chronological order, in which they play a role in the EJC’s life cycle.

## 1. Introduction

**Figure 1:**
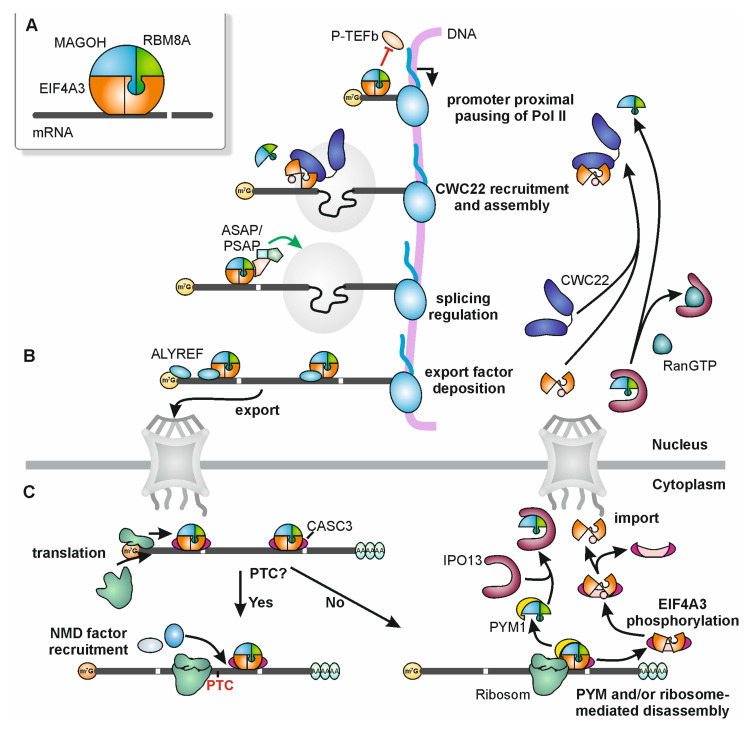
Exon junction complex (EJC) lifecycle. (**A**) Schematic representation of the trimeric EJC consisting of EIF4A3 and RBM8A–MAGOH. (**B**) Starting in the nucleus, the EJC escorts mRNAs and fulfills multiple functions regulating mRNA expression. Non-canonically deposited EJCs enhance promoter proximal pausing of RNA polymerase II (Pol II) by preventing Pol II association with P-TEFb. Canonical EJCs are recruited to the spliceosome via the interaction between CWC22 and EIF4A3 and are deposited 24 nucleotides (nts) upstream of an exon-exon junction. These EJCs can influence splicing via various mechanisms (see Figure 2 for details; ASAP: ACIN1-RNPS1-SAP18; PSAP: PNN-RNPS1-SAP18) and can stimulate the deposition of RNA export factors (e.g., ALYREF) onto the mRNA (see Figure 3 for details). (**C**) Before, during or after transport of the EJC-associated mRNAs to the cytoplasm, the EJCs undergo a compositional switch (see Figure 4 for details) and are removed from the mRNA during translation by ribosomes that can be equipped with PYM1 or by PYM1 alone. When a premature translation termination codon (PTC) is located sufficiently upstream of an EJC, this EJC is not removed during translation and therefore can induce nonsense-mediated mRNA decay (NMD) by recruiting NMD factors (see Figure 4 for details). EJCs that are removed by PYM1 are disassembled into the RBM8A–MAGOH heterodimer and the remaining EIF4A3 and CASC3. EIF4A3, which is in its open conformation, can be phosphorylated by CDK1/2 and re-imported to the nucleus by an unknown mechanism. The RBM8A–MAGOH heterodimer is imported to the nucleus by Importin 13 (IPO13).

The exon junction complex (EJC) is a multi-protein complex and universal component of spliced messenger ribonucleoproteins (mRNPs) in metazoans that carries out important functions in mRNA metabolism (reviewed in [1,2,3]). EJCs consist of three invariable core factors (Figure 1A) and many interacting proteins, which vary depending on cellular context and gene expression step. The assembly of EJCs during intron splicing results in their binding to mRNAs approximately 20–24 nucleotides (nts) upstream of exon–exon junctions in a sequence-independent manner [4,5]. Due to their remarkably stable interaction with RNA [6,7], EJCs accompany a bound mRNA during its entire lifecycle. Accordingly, the EJC core (Figure 1A) and EJC-interacting proteins are involved in almost all steps of gene expression (reviewed in [1,2,3]) (Figure 1B). While some of the EJC-mediated functions have been studied and described in great detail, others are only poorly understood. Based on the work of the past years we have now gained a more complete impression of the life and activities of the EJC. A picture emerges, in which the formation of the EJC has immediate effects on the integrity and correct maturation of mRNAs [8,9] (Figure 1B). In addition, the presence of the EJC indirectly affects mRNA export, translation and turnover [10,11,12] (Figure 1B,C). This review summarizes the course of a day in the fascinating life of the EJC—starting in the “morning” with its spliceosome-assisted assembly to the “evening”, when the EJC is disassembled into its individual parts in the cytoplasm. Due to the complexity of the topic, we will not be able to describe all phases of the EJC’s life in great detail. Instead, our goal is to provide a balanced general overview with a focus on new developments of the recent years.

## 2. Components of the EJC

Three core proteins are required to form of a stable EJC during splicing: EIF4A3, RBM8A (a.k.a. Y14) and MAGOH (two homologous proteins MAGOH and MAGOHB expressed from two genes in mammals) [13,14]. The DEAD-box protein EIF4A3 is the main RNA-binding component. RBM8A and MAGOH form a stable heterodimer, which locks EIF4A3 in its RNA-bound conformation on the mRNA and inhibits the ATPase activity of EIF4A3 [6,7] (Figure 1A).

For many years, the protein CASC3 (previously referred to as MLN51, BTZ or Barentsz) was considered the fourth EJC core component due to its stable interaction with EIF4A3 [13,15] and its requirement for the assembly of recombinant EJCs in vitro [16]. Moreover, CASC3 was also present in the crystal structure of the EJC [6,7]. However, there had been recurrent discussions about the exact role of CASC3 in the EJC. What sparked off this discussion was the fact that at steady state CASC3 does not localize in the cell nucleus and to nuclear speckles like the other EJC core components, but is mainly found in the cytoplasm [17]. However, CASC3 shuttles between the nucleus and the cytoplasm and co-localizes with other EJC components in the nucleus if its export is inhibited [18]. Furthermore, CASC3 has been reported to be much less abundant than the other EJC core proteins in the cell, suggesting that it may not interact constantly with all EJC cores [19]. Two recent publications now provided further evidence that CASC3 is not an obligate component of all EJCs. One of these papers reported that soon after their assembly, EJCs exist within higher-order mRNPs, which also contain splicing factors such as RNPS1, SR (serine/arginine-rich) and SR-like proteins, but not CASC3 [11] (Figure 1B). Later in their life, around the time of their export to the cytoplasm, EJCs undergo a compositional switch and exist mainly as CASC3-containing monomeric EJCs (Figure 1B,C and Figure 4). In the second publication, CASC3 knockout (KO) cells were generated and the effects of CASC3 deficiency on EJC assembly and function were analyzed [20]. In contrast to the essential function of CASC3 during the assembly of recombinant EJCs in vitro [16], the absence of CASC3 did not quantitatively or qualitatively affect the assembly of EJCs during splicing in living cells [20]. Furthermore, the nuclear functions and the overall composition of the EJC were virtually unchanged. However, the CASC3 KO cells showed a defect in the turnover of mRNAs that are substrates of nonsense-mediated mRNA decay, an EJC-dependent quality control pathway that is described in detail below. Although the two publications differ in their weighting and interpretation of CASC3′s function in NMD, the common denominator is that CASC3 does not play a role during the early life of the EJC in the nucleus.

Immediately after their synthesis in the cytoplasm, RBM8A and MAGOH assemble into a heterodimer, which is transported by importin 13 (IPO13) to the nucleus [21,22] (Figure 1C). Although the heterodimer likely represents the biologically predominant form, RBM8A has also been described to have EJC-independent functions, for example as a factor involved in DNA damage repair and modulator of processing body formation [23,24]. Still, it remains to be investigated to what extent RBM8A and MAGOH have functions separate from each other or as a heterodimer outside the EJC. Likewise, EIF4A3 is also imported into the nucleus, but in contrast to RBM8A–MAGOH no further details about this important step for EJC assembly are known. After import of the individual components, they are assembled into the core EJC during splicing (Figure 1B). Several so-called peripheral EJC proteins can then interact with the newly formed EJCs (reviewed in [1,2,3]). It seems reasonable that EJC-interacting proteins have to meet certain criteria to be called peripheral EJC components. We therefore propose to restrict the term to those proteins or protein complexes that specifically interact with the complete EJC core in its mRNA-bound form. This excludes proteins such as CWC22 that interact with individual, unassembled EJC core proteins. The same applies to many spliceosomal proteins that were found as EJC interactors by mass spectrometry [19]. Given their function and that their interaction with the EJC occurs inside the spliceosome, we would describe these proteins as EJC assembly factors [19]. Peripheral EJC components are thought to bind to the EJC core whenever they are needed to mediate the various functions of the EJC. Some of the peripheral EJC proteins themselves form complexes, which interact with the EJC [25]. Functionally, most peripheral EJC proteins can be classified as either regulators of splicing, export or nonsense-mediated mRNA decay. The most important representatives of these peripheral proteins and complexes are the ASAP (ACIN1-RNPS1-SAP18) and PSAP (PNN-RNPS1-SAP18) complexes [8,9,25,26], the TREX complex including the export factor ALYREF (previously known as Aly or THOC4) [12,27] and the NMD protein UPF3B [28]. Of these, only the interaction of the EJC with UPF3B via its exon-junction complex binding motif (EBM) is structurally understood [28]. Insight into the UPF3B-EJC interaction has led to the identification of several other putative EJC-interacting proteins containing EBMs [29]. For a better understanding of the interaction and function of peripheral EJC proteins additional structures of EJC-containing complexes are needed.

## 3. Spliceosomal EJC Assembly

EJCs bind to mRNAs at specific sites approximately 20–24 nts upstream of the spliced exon-exon junction [4,5]. The observation that this binding is independent of the sequence context and occurs at a specific position relative to the spliced-out intron has puzzled researchers for many years. In 2012, it was reported that EIF4A3 directly interacts with the MIF4G domain of the spliceosomal protein CWC22, providing a simple and logical explanation for the intimate coupling of EJC deposition and pre-mRNA splicing [30,31,32,33] (Figure 1B). Although it is not known if CWC22 and EIF4A3 are recruited together to the spliceosome, studies with purified proteins demonstrated that they can form a complex outside the spliceosome [30,31]. Within the EIF4A3–CWC22 dimer, EIF4A3 adopts an inactive conformation that prevents premature RNA binding [34]. Hence, in order to interact with RBM8A–MAGOH and with RNA, EIF4A3 has to undergo a conformational change accompanied by ATP binding. Whether the conformational change of EIF4A3 is imposed by MAGOH–RBM8A or by the rearrangement of the spliceosome during splicing remains elusive. Likewise, the molecular details of the RBM8A–MAGOH recruitment to the spliceosome are not yet understood.

Spliceosome assembly follows a strict choreography through a series of spliceosomal complexes [35]. Initially, binding of U1 and U2 small nuclear ribonucleoproteins (snRNPs) to the 5′ splice site and the branch point, respectively, leads to the formation of the spliceosomal A complex. Subsequent recruitment of the U4/U6.U5 tri-snRNP results in the B complex, which is converted through compositional and structural rearrangements into the activated spliceosome (B^act^ complex). The catalytically active B* complex catalyzes the first step of splicing, resulting in the formation of the spliceosomal C complex. Finally, the intron is excised and the exons ligated together during the second step of splicing.

The splicing factor CWC22 was initially identified as component of activated spliceosomes, but its central function for EJC assembly was not known [36]. Cryo-electron microscopy (Cryo-EM) structures have now provided insights into the interaction network of the EJC core within the spliceosome. Within spliceosomal B^act^ complexes, CWC22 occupies a position, from which it can guide the assembly of the EJC upstream of exon-exon junctions like a molecular ruler (reviewed in [37]). B^act^ complexes only seem to contain CWC22, but not EIF4A3 or any other of the EJC core proteins [38,39]. This suggests that CWC22 binds to the spliceosome initially alone and EIF4A3 follows later. However, it is also possible that the presence of EIF4A3 in B^act^ spliceosomes is missed for technical reasons. In contrast, EIF4A3 is found to interact with CWC22 in spliceosomal C, C* and P complexes even after the EJC is fully assembled [36,40]. The recently reported interaction between the splicing factor CWC27 and CWC22 adds additional details to the assembly pathway of the EJC [41]. Accordingly, the CWC27/CWC22 heterodimer may serve as a landing site for EIF4A3 in the B^act^ spliceosome. Shortly afterwards, CWC27 leaves the spliceosome before the EJC is assembled. This avoids a possible clash between RBM8A and CWC27 whose simultaneous presence in the spliceosome would not be compatible and suggests a step-wise pathway for the assembly of the EJC involving an CWC27/CWC22/EIF4A3 intermediate. In addition to CWC22, the spliceosomal U5 snRNP component EFTUD2 forms a direct contact with EIF4A3 as part of the assembled EJC in the spliceosomal C complex (reviewed in [42]). This interaction could support the binding of the 5′ exon to the spliceosome during the second step of splicing, but is probably not required for EIF4A3 recruitment to the spliceosome or EJC assembly. The intron-binding helicase AQR (a.k.a. IBP160, aquarius) has been reported to be responsible for the initial binding of EJC components to the spliceosome [43]. However, the contribution of AQR to EJC assembly requires further investigation, as AQR is located on the opposite side of the B^act^ spliceosome, compared to CWC22 and EIF4A3 [39]. Notably, CWC22 does not only mediate EJC assembly by the spliceosome. It is also directly involved in the splicing process and interacts extensively with PRPF8, the catalytic center of the spliceosome [33,36,40]. Nevertheless, the precise role of CWC22 in pre-mRNA splicing remains to be determined.

As described above, there is growing evidence that CASC3 has no function during the assembly of the EJC [11,20]. However, CASC3 is detected bound to the EJC core in all spliceosomal structures reported so far. This apparent discrepancy could be explained by the fact that the spliceosomes used for the structural studies were assembled in vitro in nuclear extracts, which may facilitate the interaction with CASC3 present in the extracts. Alternatively, a so far unidentified protein could interact with the CASC3 binding site of EIF4A3 within the spliceosome in a similar way as CASC3. Indeed, there are spliceosomal proteins that contain sequences that are homologous to the EIF4A3 binding sequence of CASC3 [27]. However, in order to differentiate between these possibilities, structures of the spliceosome with quasi-atomic resolution would be required.

The positions of the EJC on its bound mRNA have been investigated in studies using CLIP (crosslinking and immunoprecipitation of RNA–protein complexes), RIP (RNA immuno-precipitation) and related techniques. These revealed that the EJC does not only bind to the canonical deposition site, 20–24 nts upstream of an exon-exon junction, but also at other positions in exons [5,19]. In mammals, these non-canonical binding sites are quite frequent, ranging up to 50% [5], while in *Drosophila* nearly all EJCs seem to be deposited at canonical binding sites [4]. Interestingly, CASC3 containing EJCs also show a strong bias towards canonical deposition, further supporting the assumption that it is not present in all EJC cores [44]. Additionally in human cells, different studies display differences in their proportion of canonical and non-canonical EJC deposition [45]. But it is reasonable to assume that the technical details of the experiments, e.g., whether translation inhibitors were used or not, may have a major influence on the apparent localization of EJCs.

## 4. Splicing Regulation by the EJC

**Figure 2:**
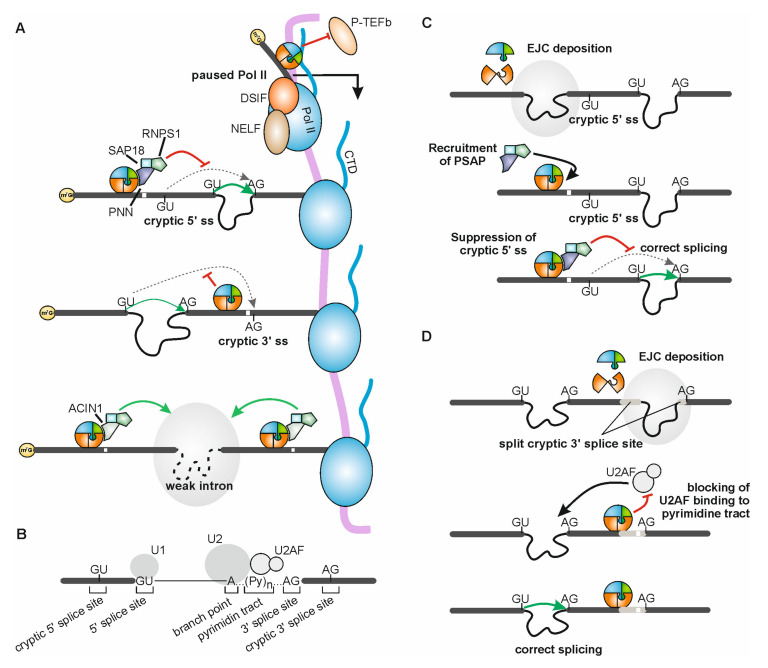
Mechanisms of splicing regulation by the EJC. (**A**) Non-canonical EJCs can bind to the mRNA at promoter regions and interact with the C-terminal domain (CTD) of paused Pol II, which is stabilized by DRB sensitivity-inducing factor (DSIF) and negative elongation factor (NELF). This prevents binding of the transcription elongation factor P-TEFb and thereby holds the Pol II in a paused state, which ensures proper splicing. Additionally, EJCs deposited at canonical sites enhance splicing of neighboring weak introns in *Drosophila*, potentially via the ASAP complex (ACIN1, depicted in grey-green color; SAP18, RNPS1). Furthermore, the EJC core represses the usage of reconstituted cryptic 3′ splice cites by steric hindrance. RNPS1 as a PSAP (PNN, depicted in violet; SAP18; RNPS1) component represses cryptic 5′ splice sites located downstream of the EJC. (**B**) Schematic representation of intronic sequences involved in splicing. The 5′ splice site is bound by the U1 snRNP, the branch point by the U2 snRNP. U2AF (U2AF1/U2AF2) recognize the pyrimidine tract and help recruiting the U2 snRNP. Cryptic 5′ and 3′ splice sites in exons resemble authentic splice sites, but are located outside the intron. (**C**) Stepwise model of cryptic 5′ splice site repression by the EJC and the PSAP complex. In a first step, the EJC is deposited to an upstream exon-exon junction during splicing. Recruitment of RNPS1 as a PSAP component is sufficient to suppress usage of a nearby cryptic 5′ splice site and thereby enables correct splicing of the downstream intron. (**D**) Stepwise model of cryptic 3′ splice site repression by the EJC core. Splicing of the downstream intron reconstitutes a cryptic 3′ splice site that is split across two neighboring exons. During splicing, the EJC is deposited at the exon-exon junction and thereby masks the reconstituted cryptic 3′ splice site. The U2AF splice factor is therefore unable to bind to the pyrimidine tract of the cryptic splice site and splicing of the upstream intron is enforced.

Once the EJC is deposited onto the mRNA at an exon-exon junction, it can influence the expression of the bound mRNA in various ways. A particularly interesting example is the regulation of certain splicing events, which was described in *Drosophila melanogaster* for the first time in 2010 [46,47]. Shortly thereafter, EJC-regulated splicing was also documented in human cells. The depletion of EJC proteins caused a wide range of different splicing effects, including the use of alternative 5′ and 3′ splice sites; exon inclusion and exon skipping; and the retention of short introns [48,49]. However, some of the underlying mechanisms have only recently been elucidated and seem to be substantially different between humans and flies (Figure 2). Whether the molecular mechanism is determined by factors involved in the splicing process or by the differing architecture of the pre-mRNAs in the two organisms remains to be investigated. Further analyses in other organisms could provide hints about the possible reasons for the reported differences.

In human cells, the presence of the EJC prevents re-splicing of 5′ and 3′ splice sites located within exons, the so-called cryptic splice sites [8,9] (Figure 2A,B). EJC-suppressed cryptic splice sites resemble canonical splice sites, but are hardly used under normal circumstances (i.e., in the presence of an EJC); 5′ splice sites that are suppressed by the EJC are usually located within the first 40 nts or directly at the beginning of an exon. Usage of these 5′ splice sites in the absence of the EJC leads to the removal of the exonic sequence downstream of the 5′ splice site. Depending on the exact position of the 5′ splice site, its use resembles exon skipping or splicing at an alternative 5′ splice site. Mechanistically, the EJC prevents the undesirable re-splicing of cryptic 5′ splice sites by recruiting the PSAP complex, an EJC associated protein complex consisting of RNPS1, SAP18 and PNN (Figure 2A,C). In this case, RNPS1 is the effector molecule that, when located at short distance upstream of the cryptic 5′ splice site, inhibits splicing at this site. For this function only the central RNA recognition motif (RRM) of RNPS1 is required, but neither of its other domains. The RRM of RNPS1 is also sufficient for the assembly of the ASAP or PSAP complex [26]. Since the other PSAP components cannot repress cryptic splice sites in the absence of RNPS1 [9], the RRM probably interacts with further protein factors in addition to SAP18 and PNN. Notably, RNPS1 has also been described to protect the AURKB mRNA from cryptic splicing independently of the EJC [49]. The obvious similarity of the reported effects on cryptic splice sites indicates that EJC-dependent and independent effects of RNPS1 follow a similar molecular mechanism.

In contrast to 5′ splice sites, which normally only consist of a 5 nts consensus sequence, 3′ splice sites are composed of a longer sequence, to which the branch point, a pyrimidine tract and the actual 3′ splice site (AG) belong (Figure 2B). It was shown that EJC-suppressed cryptic 3′ splice sites can be formed by a splicing event, in which the pyrimidine-tract of an upstream exon is joined with the actual 3′ AG splice site in the downstream exon [9] (Figure 2D). Cells lacking EJC core components exhibit increased usage of these reconstituted cryptic 3′ splice sites, leading to a substantial loss of exonic sequences. The presence of the EJC blocks splicing presumably by masking the 3′ splice site so that it cannot be recognized by the splicing factor U2AF. This mechanism also explains the high efficiency by which EJCs prevent the usage of cryptic 3′ splice sites, since any EJC deposited during the first splicing event would have to be removed before U2AF can bind to its recognition site [9].

In *Drosophila*, the EJC is required for splicing of intron 4 in the PIWI pre-mRNA and a number of additional introns [50,51]. Deposition of EJCs at upstream and downstream exon-exon junctions mediated by splicing of intron 3 and 5, respectively, supports the recognition and efficient splicing of the PIWI intron 4 [50] (Figure 2A). For this function of the EJC, RNPS1 and Acinus appeared to be necessary. In contrast, neither SAP18 nor PNN were involved in splicing of PIWI intron 4, which implies that in spite of their similar composition, ASAP and PSAP complexes have different functions in splicing regulation. The fact that SAP18 was not required in this context even suggests that a dimeric ASAP complex lacking SAP18 exists that is able to conduct splicing regulation [51]. A recent study using human cell culture indicates that also in mammals ASAP and PSAP complexes are functionally different [25]. In the absence of PNN and consequently absence of the PSAP complex, several alternative splicing events occurred that were not rescued by ACIN1 and the ASAP complex. The functional difference between the complexes is also indicated by the distinct set of alternative splicing events regulated by them. Among these sets are also splicing events dependent on the core of the EJC, suggesting that the complexes can act either independently or in combination with the EJC core. Interestingly, both complexes seem to interact with the EJC via their individual component, ACIN1 or PNN. This might also explain why the regulation of 5′ splice sites by RNPS1 requires not only EJC deposition but also the assembly of the PSAP complex, to recruit RNPS1.

The EJC has also been shown to communicate with RNA polymerase II (Pol II) and thereby to link the processes of splicing and transcription. EJC core proteins were found to bind to intron-containing and intronless transcripts and to associate with transcribed genes in a splicing-independent manner in *D. melanogaster* [52]. Recently, it was shown that EJC components are recruited at promotors without prior splicing and prevent exon skipping in large intron-containing transcripts by regulating promotor proximal pausing of Pol II. Specifically, binding of the EJC to nascent mRNAs at the promoter regions stabilizes Pol II in a paused state [53] (Figure 2A). This effect is mediated by restricting the association of the positive transcription elongation factor (P-TEFb) complex with Pol II. By phosphorylation of the negative elongation factor (NELF) and Ser2 of Pol II, the P-TEFb complex promotes the release of Pol II from its paused state. If the EJC is missing, P-TEFb can immediately bind to Pol II and initiate the elongation phase of transcription. This leads to frequent exon skipping events, suggesting that the promoter proximal pausing of Pol II, regulated by splicing-independent recruitment of EJCs, is an efficient and necessary step to ensure proper splicing. Additionally, in human cells a recent publication suggests that the EJC modulates Pol II transcription. In brief, the splicing of internal exons was shown to influence promoter choice, an effect referred to as exon-mediated activation of transcription starts (EMATS) [54]. Although the mechanism of this process is not fully understood, high-throughput data indicate that it may be—at least in part—mediated by the EJC in conjunction with other RNA-binding proteins.

## 5. EJC and mRNP Export

**Figure 3:**
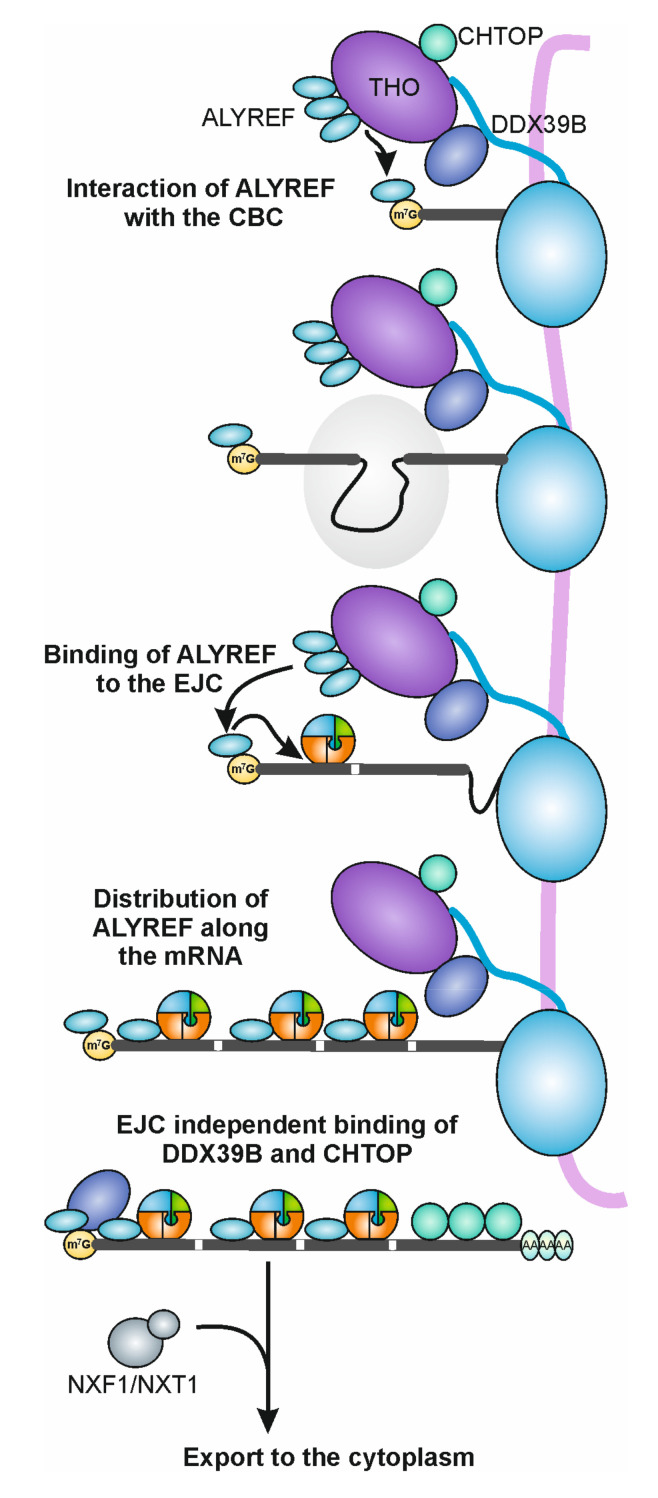
Recruitment of mRNA export factors by the EJC. The TREX complex (THO, DDX39B, ALYREF) interacts with the CTD of Pol II. The mRNA export factor ALYREF is first recruited to the CBC and in a next step distributed on the mRNA upstream of canonical EJC positions by an interaction with the EJC component EIF4A3. DDX39B and CHTOP bind in an EJC-independent manner to the cap binding complex (CBC) and the 3′ UTR of the mRNA, respectively. When all export factors are deposited and polyadenylation is completed, the export receptors NXF1/NXT1 are recruited and promote export of the mRNA.

Another function of EJC in the cell nucleus is to ensure proper export of mRNAs to the cytoplasm by facilitating the association of export factors with mature, completely processed mRNAs (Figure 3). To fulfill this function, another multiprotein complex is required, the transcription/export (TREX) complex. As the name suggests, the TREX complex couples transcription and export of mRNAs [55]. It is composed of several components, including the THO subcomplex, the DEAD-box protein DDX39B, mRNA export adaptors and co-adaptors such as ALYREF and CHTOP, respectively (reviewed in [56]) (Figure 3). While previous studies reported that the cap binding complex (CBC) recruits and links ALYREF and the TREX complex to the mRNA [57], a recent publication suggested that this represents an intermediate step in the assembly of export-competent mRNPs [12]. Instead, it appears that ALYREF is initially recruited by the CBC, but its subsequent binding along the mRNA also relies on splicing and the deposition of EJCs (Figure 3). An alternative model exists, in which ALYREF is mainly recruited to the 5′ and 3′ ends of the mRNA via its interaction with the CBC and the 3′ processing factor CstF64, respectively [58]. The recruitment of ALYREF by the EJC is likely mediated through an interaction with EIF4A3, to which it binds via a short peptide sequence that is homologous to the EIF4A3-binding sequence in CASC3 ([27], and reviewed in [42]). This is supported by the observation, that ALYREF binds to the mRNA at positions directly upstream of EJC binding sites [12]. CHTOP on the other hand, which is not known to interact with the EJC, is deposited mainly on the last exon and in the 3′ UTR (Figure 3). Interestingly, both factors are deposited before 3′ end processing of the RNA [12]. Hence, it remains unknown how the actual export of the mRNA is initiated, given that the export adaptors are already bound to the mRNA before it is fully processed and ready for export. In general, the export function of the EJC is not yet fully understood and it will be important to distinguish between direct and indirect effects of the EJC on mRNA export.

## 6. Function of the EJC during mRNA Translation

It has been shown in several organisms and with different experimental approaches that spliced mRNAs are expressed at higher levels and produce more protein product than unspliced mRNAs [59]. The higher expression rate may be due to more efficient 3′ processing, escape from nuclear decay pathways, enhanced mRNA export or increased cytoplasmic stability. But even if all these effects are subtracted, spliced mRNAs are still better translated than unspliced mRNAs [60]. Using different experimental approaches, the EJC was shown to be responsible for this effect. Three different molecular mechanisms mediated by the proteins PYM1, SKAR and CASC3 were described.

The protein PYM1 (partner of Y14-MAGOH, previously referred to as PYM or WIBG) was suggested to bridge the EJC to the 48S preinitiation complex and enhance the translation of spliced mRNAs. In accordance with this suggestion, it was shown that PYM1 pulls down protein components of the large and small ribosomal subunits in immunoprecipitations. Furthermore, the knockdown of PYM1 reduced the translation yield of spliced reporter mRNAs. However, it should be noted that PYM1 does not bind to the full EJC, but only to the RBM8A–MAGOH heterodimer [61,62]. Within the trimeric PYM1–RBM8A–MAGOH complex, the RBM8A–MAGOH heterodimer cannot bind to EIF4A3. Due to a steric clash between EIF4A3 and PYM1, they bind to RBM8A–MAGOH in a mutually exclusive manner. This clash is likely the reason why PYM1 acts as an EJC disassembly factor, as was shown later. Thus, it remains to be examined whether and how these two functions of PYM1 are compatible with each other. SKAR (S6K1 Aly/REF-like target; POLDIP3) is another EJC-associated protein that was proposed to mediate the stimulatory effect of EJCs on mRNA translation [63]. As a known S6K1 substrate, SKAR is thought to recruit S6K1 to EJC-bound mRNAs and to transmit mTOR signals to newly made mRNPs. Indeed, several mRNP components were found to be phosphorylated by S6K1 in an EJC-dependent manner. However, it is not completely understood which phosphorylation of what proteins leads to the upregulation of translation. The third EJC-bound protein that was shown to function as a translation stimulator is CASC3 [10]. Knockdown and overexpression of CASC3 decreased and increased, respectively, the translation efficiency of reporter mRNAs. Although this effect was also observed on intronless mRNA, it was much more pronounced on intron-containing, spliced mRNA carrying an EJC. In addition, CASC3 was shown to interact with the initiation factor eIF3 via its SELOR domain. It was suggested that eIF3 is recruited via the EJC and CASC3 to spliced mRNAs as soon as they enter the cytoplasm and enhances their translation.

Whatever the mechanism might be by which the EJC increases the translation of spliced mRNAs, it appears to aid the translation machinery to distinguish newly formed mRNAs from transcripts that have already been translated. Potentially, this mechanism was originally designed to support the production of proteins from genes whose transcription has recently been switched on (reviewed in [64]).

## 7. EJC-Dependent Nonsense-Mediated mRNA Decay

Nonsense-mediated mRNA decay (NMD) is a eukaryotic quality control mechanism that detects and degrades mRNAs with premature translation termination codons (PTCs) and other anomalous termination events (for details please read the review [65]). Thereby NMD prevents that C-terminally truncated proteins are expressed from mRNAs that contain nonsense mutations. However, NMD not only eliminates mutated mRNAs, but also regulates the expression of many regular mRNAs and mRNA isoforms that contain stop codons at positions that are recognized as irregular by the NMD machinery (reviewed in [66]). These so-called endogenous NMD substrates are generated for example by alternative splicing.

**Figure 4:**
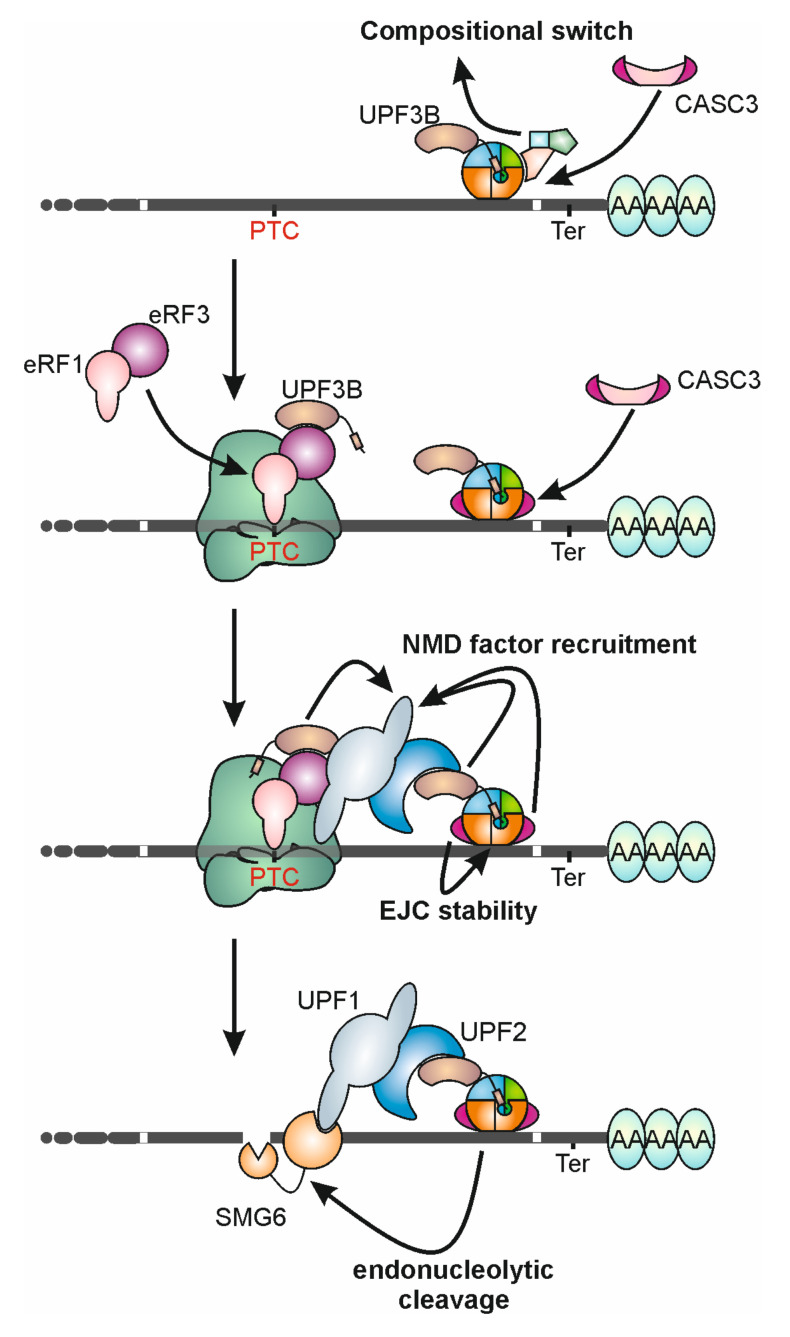
The EJC activates NMD by recruiting NMD factors. Around the time of mRNA export to the cytoplasm, the EJC undergoes a compositional switch which replaces the ASAP or PSAP complex by CASC3. This step is necessary to enable cytoplasmic EJC functions; for example, the activation of NMD. UPF3B interacts with eRF3a via its N-terminal half and with the EJC via its C-terminal EJC-binding motif (EBM; small rectangle). EJCs located downstream of a PTC will not be removed by the translating ribosomes and remain bound to the mRNA. CASC3 and the NMD factor UPF3B are then involved in the recruitment of NMD factors, such as UPF1, UPF2 and SMG6, resulting in endonucleolytic cleavage of the mRNA. For simplicity, only a small set of NMD factors is shown. Please note that the ribosome is shown upside down in comparison to Figure 1 to display all interactions as correctly as possible.

The original discovery of the EJC is tightly connected with its function in NMD in mammals. Already some time before the identification of the EJC and its main protein constituents, it was known that the splicing process and the presence of introns are required for the detection of PTCs [67,68]. This observation was explained by a mark that is deposited during splicing and tags the position of (former) introns (reviewed in [69]). Although the nature of the mark was not known at that time, it was suggested that spliceosome-associated proteins remain bound to and escort mRNAs into the cytoplasm. Shortly afterwards, the first proteins of the EJC were identified based on their ability to bind to the mRNA near exon-exon junctions [70,71,72]. At about the same time the NMD proteins UPF3A and UPF3B were described as EJC components and a model for EJC-dependent NMD was postulated [73,74]. According to this model, UPF3B bridges the EJC to the NMD machinery, because it can bind to the EJC and the NMD protein UPF2 (Figure 4). Until today this bridging model is found in variations in the literature, although in the meantime several results have been obtained that argued against this simple model. Even the exact role of the proteins UPF3A and UPF3B in NMD is the subject of controversy. Originally, both were described as functional paralogs that act as NMD activators [74]. Later it was shown that UPF3B is more potent in NMD activation due to its stronger interaction with the EJC [75]. UPF3B also readily forms a complex with UPF2 and displaces UPF3A. As a consequence, the unbound UPF3A protein becomes unstable and is hardly found to be expressed in the presence of UPF3B [76]. Thus, under normal conditions UPF3B represents the link between EJC and NMD. However, recent discoveries provided new insights into the functions of the human UPF3 proteins. First, UPF3A has been described to be an NMD inhibitor [77], although it was initially postulated to be only a weaker NMD activator than UPF3B [75]. Second, it was reported that UPF3A but not UPF3B was required for a genetic compensation response, which is triggered by gene-knockout mutations and upregulates the transcription of genes related to the inactivated gene [78]. Third, it was shown that UPF3B interacts with the eukaryotic release factor eRF3A and modulates translation termination and dissociation of post-termination ribosomal complexes [79]. This suggests that UPF3B might physically link the NMD machinery and the terminating ribosome during the recognition of a PTC (Figure 4). Given the fact that we do not know many details about these new functions of UPF3A and UPF3B, additional insights into the precise molecular mechanisms are urgently required.

Early studies showed that the peripheral EJC protein RNPS1 does not only regulate splicing, but also stimulates NMD, especially when tethered to a reporter mRNA downstream of a termination codon [80,81]. This function of RNPS1 is probably mediated by an association with NMD factors (like UPF2), but exact details of the mechanism are not known [82]. Apart from the UPF3 proteins and RNPS1, it is still unclear how the EJC contributes to the detection of PTCs. New findings now suggest that the protein CASC3 could play a more important role than previously assumed. Not only was it shown that in CASC3 knockout cells a number of known NMD substrates were upregulated but also the expression levels of many endogenous NMD-sensitive mRNA isoforms were increased in these cells [20]. However, several NMD substrates were CASC3-independent and the CASC3 KO does not have the same quantitative effects as for example the inhibition of the NMD factor UPF1. The question remains how far these results now clarify the function of CASC3. Two possible interpretations seem conceivable, which are compatible with each other. One possibility is the direct activation of NMD by EJC-bound CASC3 (Figure 4). This role of CASC3 is supported by the finding that in a tethering assay not only full length CASC3, but also its N-terminus can trigger SMG6-mediated endonucleolytic degradation of the reporter mRNA [20]. How the N-terminal sequence of CASC3 executes this function and what other factors are involved remains open. Alternatively, CASC3 may indirectly support NMD by stabilizing the EJC on the mRNA in the cytoplasm (Figure 4). Consequently, the EJC would have longer time to interact with NMD-activating proteins, leading to a global increase in NMD efficiency. Both functions of CASC3 could operate with few molecules of CASC3, which would only have to bind to a small number of EJCs that activate NMD due to their position downstream of the termination codon.

## 8. EJC Disassembly and Recycling

After the EJC has carried out its functions and accompanied its bound mRNA into the cytoplasm, it is finally disassembled and its individual components are recycled (Figure 1C). Two different pathways for EJC disassembly have been described. Translation-dependent disassembly by ribosomes targets EJCs that are located within the coding region of translated mRNAs, while translation-independent disassembly by PYM1 also removes EJC in untranslated regions [61,83]. The ability of PYM1 to associate with ribosomes suggests at least some cooperativity between these two pathways [61,84]. The mechanism of translation-dependent EJC disassembly has not been studied in great detail, but it is assumed that ribosomes simply push EJCs away from the mRNA, which are in their way during protein biosynthesis [83]. Removing the EJC from the mRNA triggers a conformational change of EIF4A3 and leads to the dissociation of the EJC. Alternatively, the ribosome may be equipped with PYM1, which interacts with and removes the RBM8A–MAGOH heterodimer from the EJC, leaving EIF4A3 and CASC3 behind [61]. PYM1 itself can also disassemble EJCs without being bound to the ribosome, which is particularly important to remove EJCs from mRNA regions that are not accessible to translation. This also seems to be the mode of action in *Drosophila melanogaster*, since *Dm*PYM is unable to interact with ribosomes at all [85]. Still, *Dm*PYM was shown to bind to Y14-MAGO and thereby is able to remove EJCs from mRNAs. Without RBM8A and MAGOH to keep it in its closed conformation, EIF4A3 adopts its open conformation and thereby is released from the RNA as well (Figure 1C). In its open conformation, EIF4A3 can be phosphorylated by cyclin-dependent protein kinases 1 and 2 (CDK1/2) in a cell cycle-dependent manner [86]. Consequently, phosphorylation of EIF4A3 (pEIF4A3) during mitosis and early G1 phase inhibits NMD in this period and dissociates EIF4A3 and CASC3 (Figure 1C). The inability to bind to the other components also has the advantageous effect that the EJC cannot spontaneously reassemble. Nevertheless, pEIF4A3 is still able to bind to CWC22, suggesting that after import to the nucleus, pEIF4A3 is guided to an active spliceosome again [86] (Figure 1B). This concludes the life cycle of the EJC and enables formation of a new EJC that resumes its function as a central component of a new mRNP.

In this review we have summarized the cellular processes that are regulated by the EJC. Apart from its molecular functions, the physiological role of the EJC is of great interest. For example, the EJC is essential for the developing nervous system of mammals and EJC components play important roles in brain development, neuronal differentiation and activity (reviewed in [87,88]). Furthermore, mutations in core and peripheral EJC proteins cause different hereditary diseases. Given the very fundamental cellular function of the EJC, its specific role in the nervous system is surprising. Therefore, it seems advisable to focus some of the future research on the activity of the EJC in neuronal cells. However, also apart from the physiological function of the EJC, many burning questions regarding aspects of its life cycle remain to be answered. These include basic questions, such as the molecular details of the import of EIF4A3 into the nucleus, but also, rather complex topics require more investigation, such as the mechanisms of NMD regulation by CASC3 and other EJC associated factors, or the details of splicing regulation by the EJC core and the ASAP or PSAP complexes. Answering these and other open questions will improve the general understanding of the EJC and how it regulates mRNA expression.
